# Supplementary material for: SDS22 coordinates the assembly of holoenzymes from nascent protein phosphatase-1
Source: Nat Commun. 2024 Jun 25;15:5359. doi: 10.1038/s41467-024-49746-4 (PMC11199634; doi:10.1038/s41467-024-49746-4)
Supplement: Supplementary file 1 — Supplementary Information [file 41467_2024_49746_MOESM1_ESM.pdf]

SUPPLEMENTARY INFORMATION

SUPPLEMENTARY FIGURES

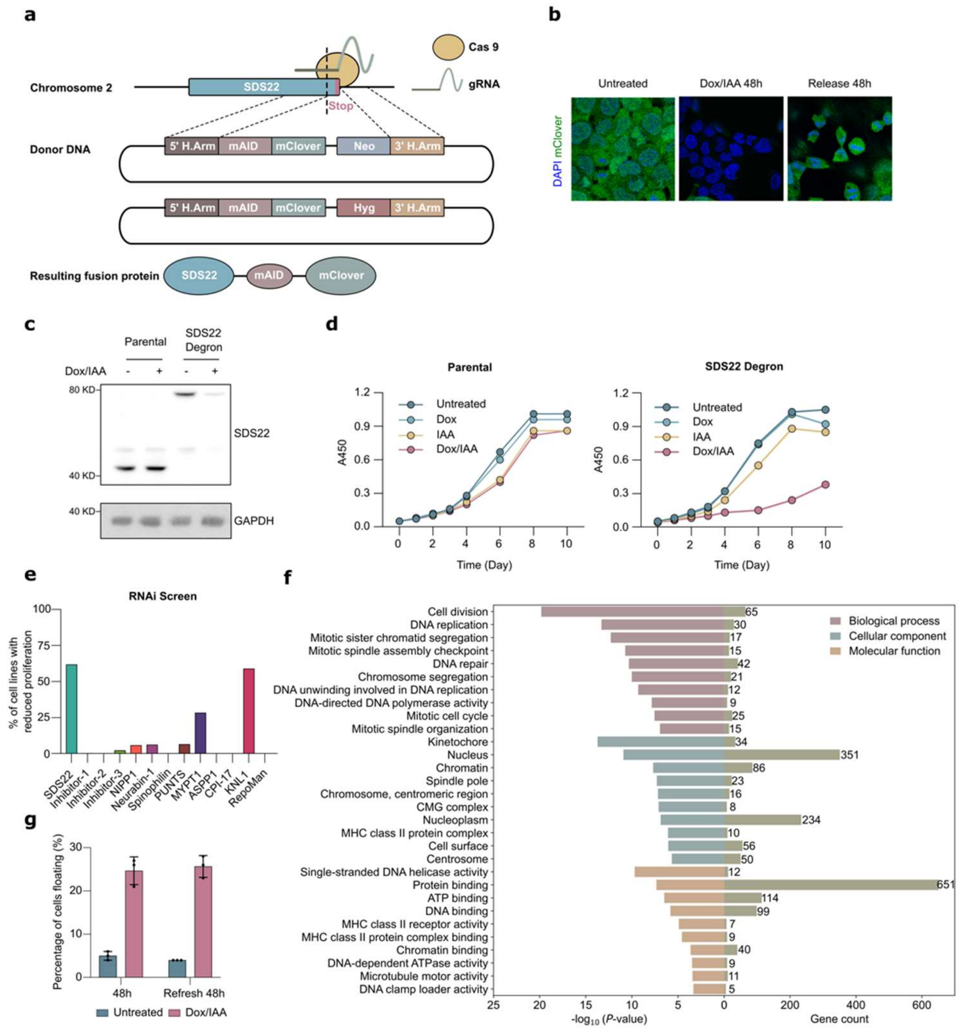

Supplementary Figure 1. Generation and characterization of SDS22-degron cell line. Related to Figure 1.

(a) Scheme of the donor DNAs used for CRISPR/Cas9-mediated modification of both *PPP1R7* alleles in HCT116 cells to express SDS22-mAID-mClover. **(b)** Expression of SDS22-mAID-mClover in the SDS22-degron cell line, without treatment (untreated), after Dox/IAA-induced SDS22 degradation (48h), or after Dox/IAA-induced SDS22 degradation (48h) plus recovery after Dox/IAA washout (48h). The fixed cells were stained with DAPI and analyzed for mClover fluorescence. **(c)** Comparison of the expression of SDS22 and SDS22-mAID-mCover in the parental and SDS22-degron cell lines, before and after Dox/IAA-treatment **(d)** Proliferation of parental and SDS22-degron cell lines (sulforhodamine B assays). The cells were untreated or treated with Dox and/or IAA. **(e)** Reduced proliferation of cancer-cell lines after the knockdown of RIPPOs, showing a major proliferation defect after the knockdown of SDS22, MYPT1 or KNL1. The data were obtained from the Dependency Map (DepMap) portal. The bars shows the percentage of 300-800 different cancer cell lines with a reduced cell proliferation. **(f)** GO analysis of differentially expressed RNAs in the SDS22-degron cells, 48h after the removal of SDS22, using the database for Annotation, Visualization and Integrated Discovery (DAVID, https://david.ncifcrf.gov/). The analysis involved 'biological process', 'cellular component', and 'molecular function'. Only the top-10 enriched pathways are shown. **(g)** Quantification of floating cells in culture medium of untreated SDS22-degron cells, Dox/IAA-treated (48h) cells, and Dox/IAA-treated (48h) + released (48h) cells, without in-between cell-splitting. The number of floating cells were determined microscopically. Floating cells were collected by centrifugation and quantified by cell counter. The results are expressed as means ± SD (n =3).

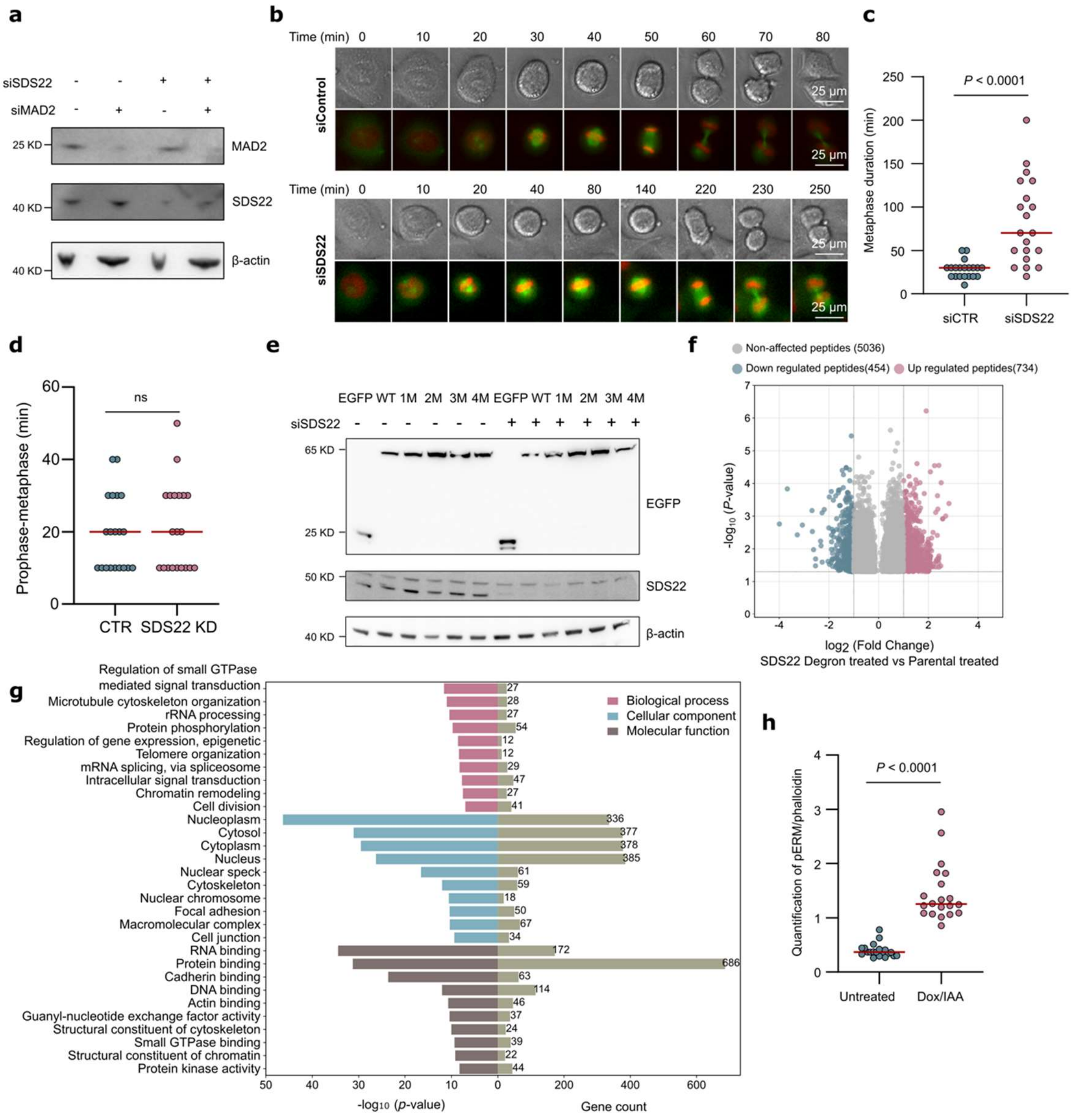

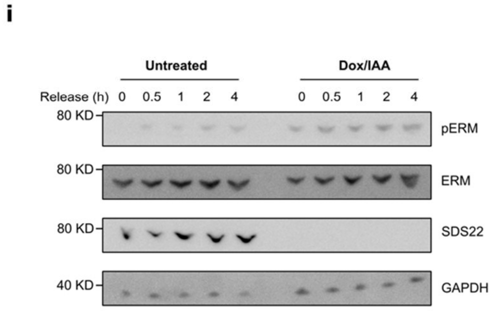

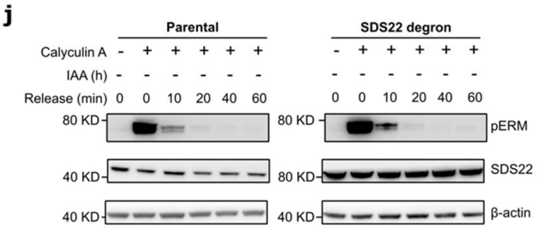

k

Gene Name	SDS22 vs parental (logFC)	*P*-value
PPP1CA	-0.040081456	0.688893
PPP1CB	-0.021163223	0.845855
PPP1CC	-0.022099949	0.830021

Supplementary Figure 2. Mitotic phenotype in SDS22-depleted cells. Related to Figure 2.
(a) Validation of the knockdown of SDS22 and MAD2 in HeLa cells. β-actin was used as loading control. **(b)** Live-cell imaging of HeLa Kyoto cells that stably expressed histone H2B-RFP and α-Tubulin-GFP. Images were taken every 10 min in synchronized cells (release from 2 mM thymidine arrest) for 48h following transfection with control or SDS22 siRNA at 0 and 24h. Green fluorescence, α-Tubuline; red fluorescence, Histone H2B-RFP; grey, bright field. Sscale bars, 25 µm. **(c)** Quantification of metaphase duration (appearance of metaphase plate → beginning of anaphase) in HeLa Kyoto cells as described in panel (b). The *P*-value is from two-sided unpaired t-test (n = 20 cells in each condition). **(d)** Quantification of prophase-metaphase duration (cell rounding → appearance of metaphase plate). The P-value is from two-sided unpaired t-test (n = 20 cells in each condition). (**e)** Transient expression of EGFP and the indicated EGFP-SDS22 fusions of SDS22 (WT; 1M: E192A; 2M: E192A + E300A; 3M: F170A + E192A + E300A; 4M: F170A + F214A + E192A + E300A) in HeLa cells, before and after knockdown of exogenous SDS22. Shown are immunoblots for EGFP, SDS22 and β-actin (loading control). **(f)** Volcano plot of altered phospho-peptides in Dox/IAA-treated (48h) SDS22-degron cells versus the similarly-treated parental cell line. Pink dots represent upregulated genes, blue dots represent downregulated genes, and grey dots represent genes that were not differentially expressed. The cutoff for significance was set at $P < 0.05$ and logFC>1.0. The data were derived from 3 independent experiments. **(g)** GO analysis of differentially detected phosphopeptides in the SDS22-degron cells, 48h after the removal of SDS22, using DAVID (https://david.ncifcrf.gov/). The analysis was focused on 'biological process', 'cellular component', and 'molecular function'. Only the top-10 enriched pathways are shown. **(h)** Quantification of pERM as shown in Figure 2f. pERM was normalized by phalloidin. The P-value is from two-sided unpaired t-test (n = 20 cells for each condition). (i) Correlation of degradation of SDS22 and ERM phosphorylation (pERM) in mitosis. SDS22 degron cells were untreated or treated with Dox/IAA for 48 h. Cells were consecutively arrested with 2 mM thymidine for 24h, released from thymidine for 4h, and arrested in nocodazole (0.1 µg/ml) for 15h. The cells were harvested at the indicated times after nocodazole release and processed for immunoblotting. **(j)** Cell-based assay for ERM dephosphorylation. Same as in Figure 2g, but for cells that were not treated with Dox/IAA. **(k)** PP1 isoform transcript level ratio (log Fc) in Dox/IAA-treated SDS22-degron cells versus parental cells, as derived from the RNA-seq data (Supplementary Figure 2C).

a

NERVIO MOTOR:	Lat [ms]	SD	Amp [mV]	SD	CV [m/s]	SD	Amp% [%]	SD	F-M [ms]	SD
Der. Peroneus										
Pos. 1 - Rec pos	2.2		2.2							
Pos. 2 - Pos. 1	4.2		4.0		50.0		84			
Pos. 3 - Pos. 2	2.2		4.5				12			
Pos. 4 - Pos. 3	4.2		5.3		50.0		17			
Pos. 5 - Pos. 4	4.2		3.8				-28			

NERVIO SENSOR:	Lat [ms]	SD	Amp [uV]	SD	CV [m/s]	SD	Amp% [%]	SD
Izq. Suralis								
Stim 1 - Rec 1	2.0		12					
Stim 2 - Rec 2	1.92		11		49.6			

Disminuir: Der. Abd pollicis brev

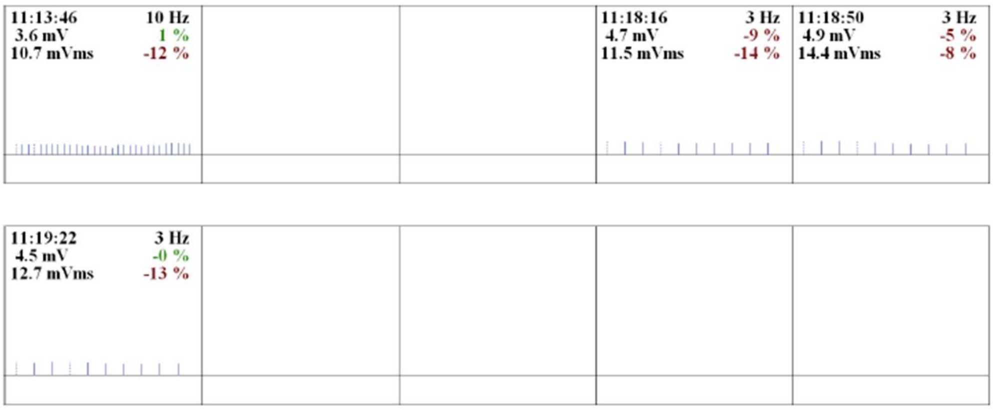

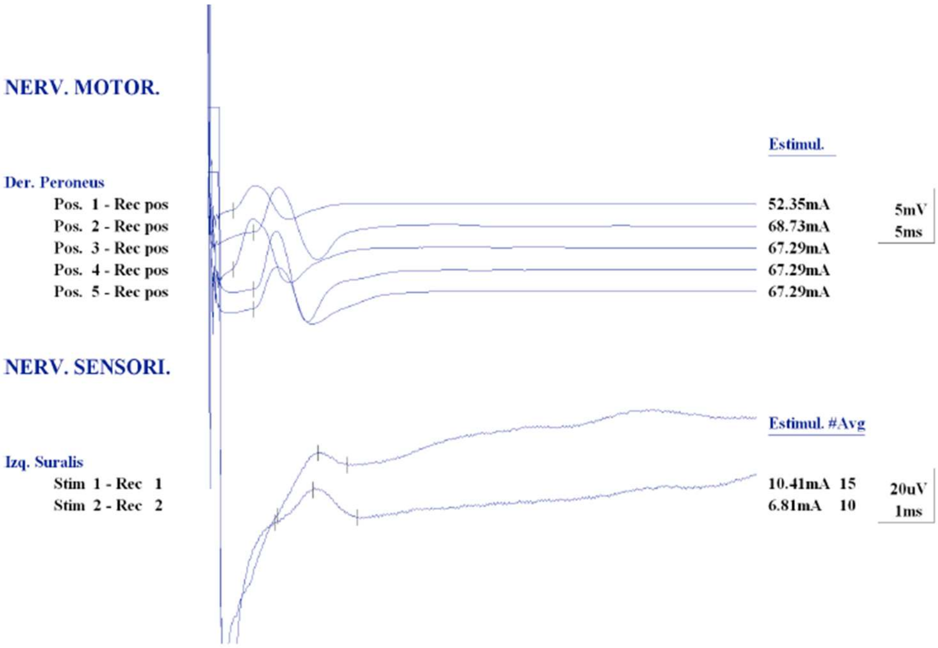

Señal. EMG

Der. Tibialis anterior (IP)

Señal. EMG

Der. Tibialis anterior (IP)

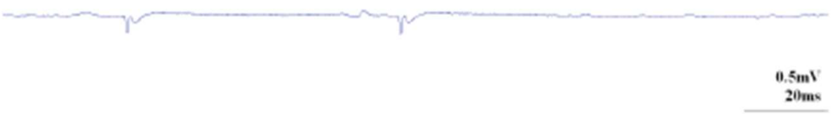

Der. Tibialis anterior (IP)

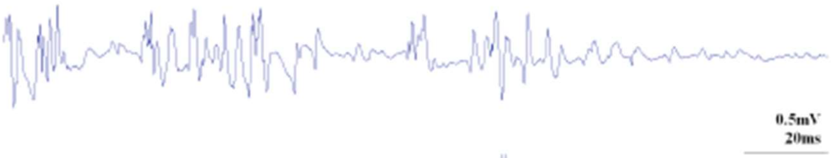

Der. Deltoideus post (IP)

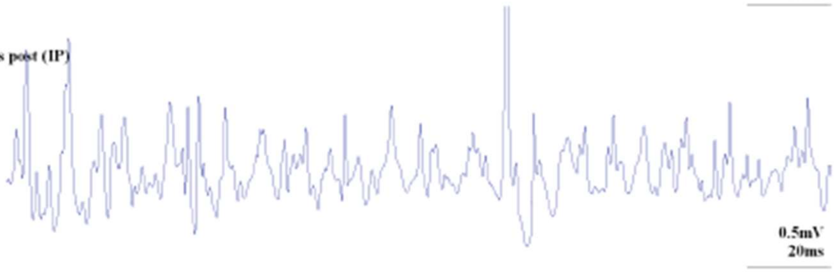

Der. Deltoideus post (IP)

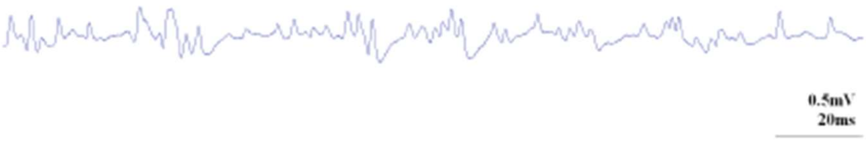

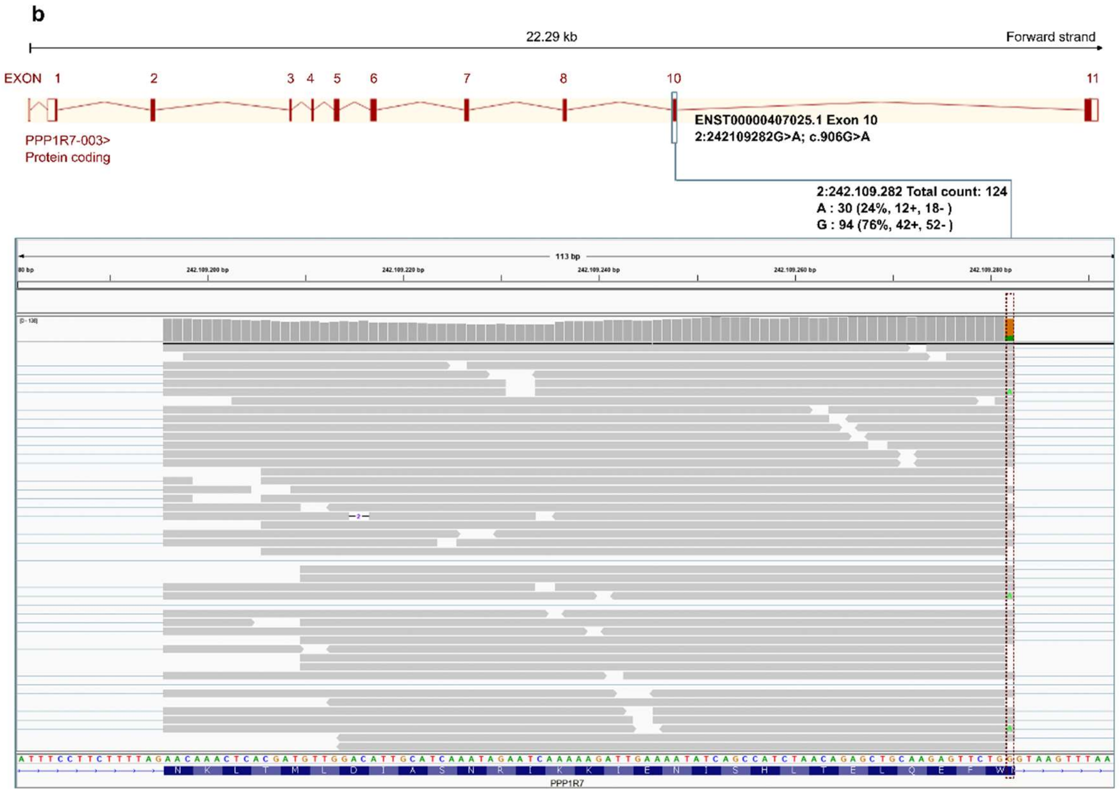

Supplementary Figure 3. Mutation of SDS22 in a patient with a neurodegenerative disease. Related to Figure 3.

(a) EMG report of patient P1. The motor and sensory neurography are within normal limits (velocities, latencies, and amplitudes in accordance with age). The electromyogram (EMG) of the deltoid and tibialis anterior muscles shows an interferential pattern with the absence of spontaneous activity at rest. No pathological decrement is observed in the repetitive stimulation test. **(b)** Mutation of *PPP1R7* (SDS22) in Patient P1. Mutation 2:242109282G>A was observed in 24% of the 124 mRNASeq reads, turning the last nucleotide of exon 10 into a stop codon (ENST00000407025.1).

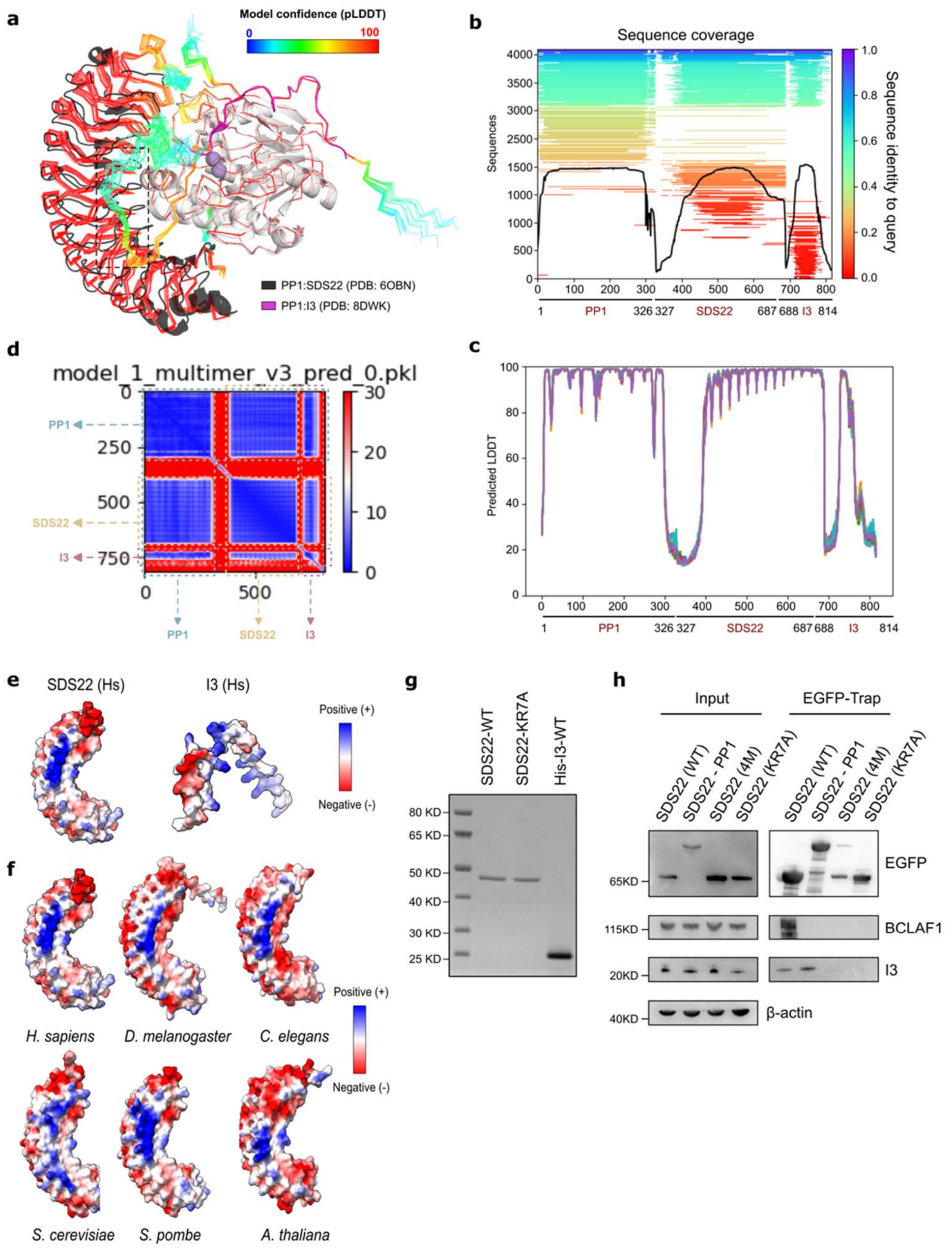

Supplementary Figure 4. Model of SDS22:PP1:I3 and conservation of the SDS22:I3 interaction interface. Related to Figure 4.

(**a**) Overlap of 25 models of SDS22:PP1:I3, as predicted by AlphaFold Multimer, using amber relaxation. Also shown are the per-residue confidence scores (pLDDT), which can vary from 0→100 (color code shown on top). Residues with pLDDT scores less than 30 are not displayed. PDB models of PP1:SDS22 (PDB:6OBN) and PP1:I3 (PDB:8DWK) were employed to validate the accuracy of AlphaFold models. **(b)** Multiple Sequence Alignment (MSA) was used as input for the network. **(c)** The pLDDT per position is given as a plot for the 25 models made in every run. **(d)** Predicted Aligned Error (PAE) gives an estimate of the relative position of domains. The predicted interacting regions are highlighted with dashed lines. **(e)** AlphaFold Multimer prediction of the electrostatic surface of SDS22 and I3. **(f)** Conservation of the basic patch of SDS22 (blue) that is predicted to interact with acidic residues of I3. *Homo sapiens* (residues 1-63 are not shown); *Drosophila melanogaster*; *Caenorhabditis elegans* (residues 1-19 are not shown); *Saccharomyces cerevisiae* (residues 1-28 are not shown); *Schizosaccharomyces pombe* (residues 1-26 are not shown); *Arabidopsis thaliana*. **(g)** Coomassie staining of purified proteins used in Figure 4k. **(h)** Co-immunoprecipitation (EGFP traps) of endogenous PP1, I3 and BCLAF1 with transiently expressed EGFP-SDS22, EGFP-SDS22-PP1, SDS22-4M (PP1-binding mutant) and SDS22-KR7A in HEK293T cells.

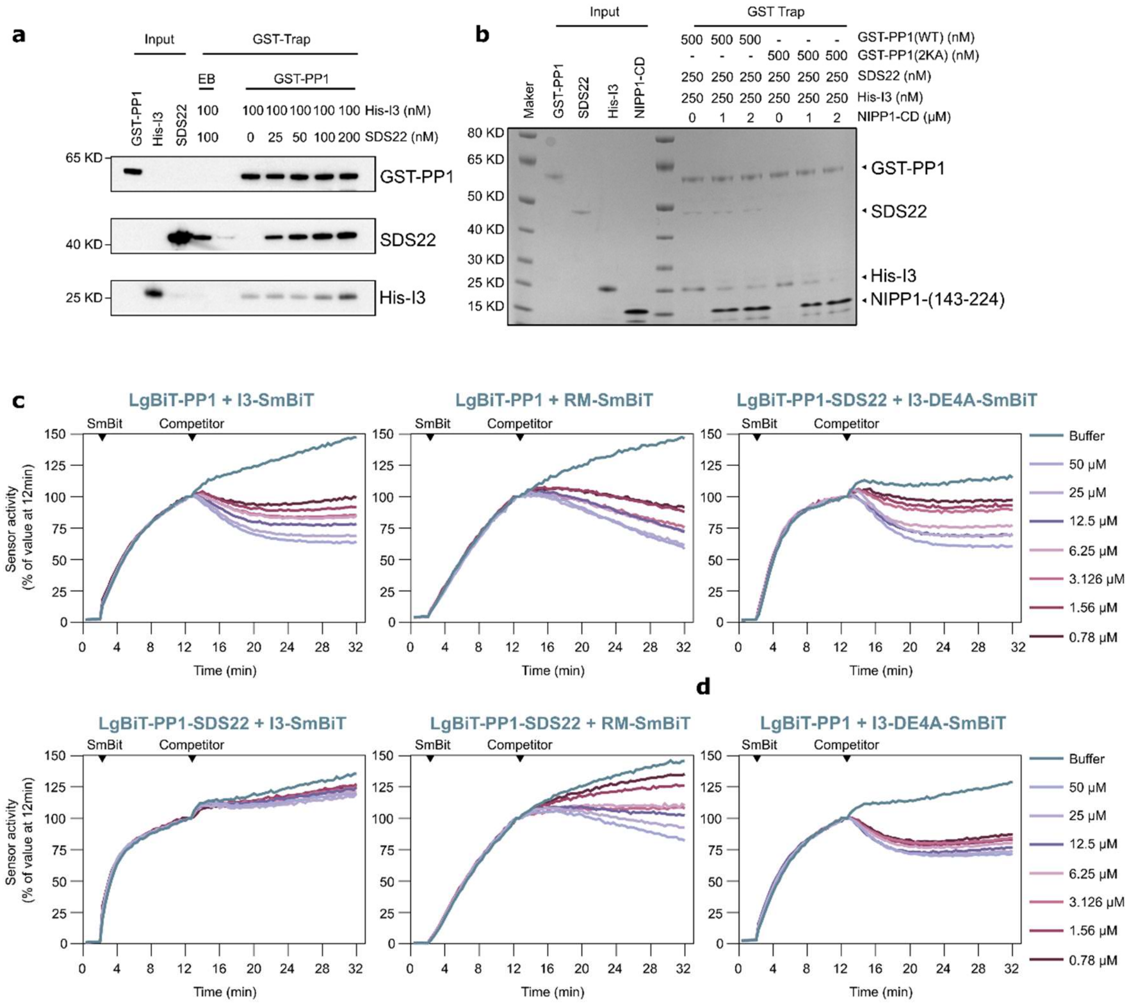

Supplementary Figure 5. The PP1:I3 interaction is enhanced by SDS22. Related to figure 5.
(a) Co-precipitation assay (GST-traps) of purified I3 with purified GST-PP1 in the presence of increasing concentration of purified SDS22. The indicated concentrations of SDS22 were incubated for 30 min at room temperature with GST-PP1 (10 μM) and glutathione magnetic beads. After washing of the beads with a buffer containing 20 mM Tris at pH 7.9 and 0.3 M NaCl, I3 (100 nM) was added. After an incubation for 15 min at RT, the beads were washed and probed for GST-PP1, SDS22 and I3 by immunoblotting. **(b)** Coomassie staining of GST-PP1-WT/2KA input and GST-traps in the presence of purified His-I3, SDS22 and the indicated concentrations of NIPP1-(143-224) competitor. **(c)** Kinetic-trace experiments as shown in Figures 5i-k, but with different concentrations (0-50 μM) of NIPP1-(143-224) competitor. The data are plotted as a percentage of the signal just before addition of competitor at 12 min. **(d)** Kinetic-trace experiment of LgBiT-PP1:I3-DE4A-SmBiT, with different concentrations (0-50 μM) of NIPP1-(143-224) competitor.

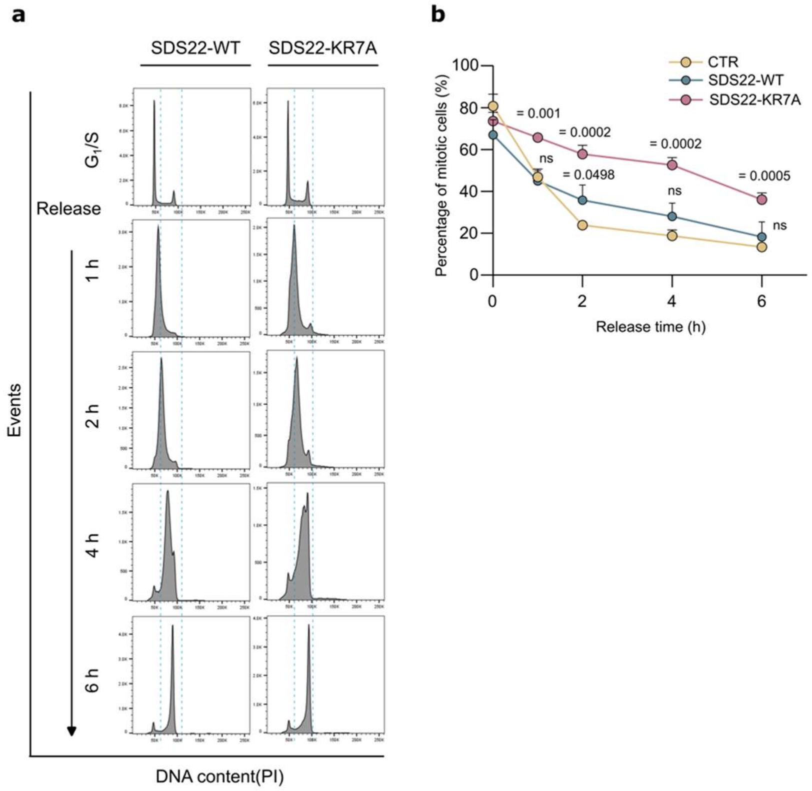

Supplementary Figure 6. Cell-cycle effects of the expression of SDS22-WT versus SDS22-KR7A. Related to Figure 6.

(a) The expression of EGFP-SDS22 or EGFP-SDS22-KR7A has no effect on S→M duration, as assessed by flow cytometry of fixed cells stained with propidium iodide, following the release from a double-thymidine arrest. **(b)** Quantification of mitotic cells after release from a G_2/M arrest, as derived from data as shown in Figure 6f. The data are represented as means ± SD; n=3 independent experiments. *P*-values are calculated by unpaired t-test.

SUPPLEMENTARY NOTE 1: Clinical data of patient P1

This supplementary note details the comprehensive clinical evaluation of patient P1. Born to non-consanguineous Caucasian parents with an uneventful pregnancy and cesarean delivery at 38 weeks, P1 exhibited hypotonia and significant delays in achieving developmental milestones from infancy. Febrile seizures occurred between 14 months and 3 years of age. Initial examination at 14 months revealed pronounced hypotonia, hyperkinesia, choreodystonic movements, ptosis, and swallowing difficulties, persisting through subsequent evaluations up to the current age of 10.

Body fluid analysis

A series of metabolic studies were normal, encompassing blood, urine, and cerebrospinal fluid analyses. Blood metabolic studies included, among others, assessments of lactate, ammonia, liver function profile, blood amino acids, sialotransferrin, very-long-chain fatty acids, glucosaminoglycans, and aminoacids (Supplementary Table 1). Aminoacids and organic acids were determined in urine (Supplementary Table 2). Cerebrospinal fluid analysis was also performed to ensure comprehensive metabolic coverage and were found unremarkable.

At the age of 8, brain magnetic resonance imaging (MRI) indicated a reduction in white matter, nerves, optic chiasm, thalami, and medulla oblongata volumes, with no signal abnormalities. Electromyography and nerve conduction velocities fell within normal ranges. A muscle biopsy at age 4 exhibited minimal, nonspecific changes. (Find below the report of MRI, electromyography and muscle biopsy).

Cranial MRI analysis of patient P1

Technique: 3T MRI using T1, T2, DP, SWI, FLAIR, and DWI sequences in all three spatial planes.
Univoxel spectroscopic study TE 144 msec in the parietal white matter. Study performed under anesthesia without complications. No previous studies available for comparison.
Prominent anterior cranial fossa. Midline structures are centered. Thinning of the chiasm and optic nerves. Hypophyseal and hypothalamic region without significant findings. Complete corpus callosum of normal thickness. Slight widening of cortical sulci accompanied by discreet increase in extra-axial spaces and slight reduction in supratentorial white matter volume, without signal alteration, all accompanied by mild dilation of the lateral ventricles.
Hippocampi without significant alterations. Adequate differentiation between white and gray matter. Normal sulcation and myelination pattern. Basal ganglia and thalamus with normal signal. Thalami appear slightly reduced in size. Brainstem shows a slight reduction in the medulla oblongata without signal alteration. Cerebellum and vermis appear normal in appearance and signal. No diffusion restriction or presence of microbleeds. Permeability of major intracranial arterial and venous structures. Permeable basal cisterns. Megacisterna magna. No descent of the cerebellar tonsils.
CONCLUSION: No evidence of vermis atrophy or signal alteration in the pons. There is a slight reduction in the volume of supratentorial white matter, including the optic nerves and chiasm, without signal alteration. Thalami appear slightly reduced in size, similar to the medulla oblongata without signal alteration. Mild dilation of the lateral ventricles, likely compensatory. Megacisterna magna. The findings described are nonspecific and do not correspond to a specific radiological pattern.

Muscular biopsy analysis of patient P1

<u>Macroscopic description</u>

A fresh sample of skeletal muscle is received, from which a small fragment is taken for fixation in glutaraldehyde and another for cryopreservation in liquid nitrogen with subsequent storage in freezing. The remaining sample is oriented transversely under a microscope and processed for cryopreservation

in liquid nitrogen. Histological sections are obtained through cryomicrotomy, followed by routine staining.

Microscopic description
The histological study reveals well-preserved muscle fibers with slight variability in fiber size without an increase in internal nuclei. Occasional fibers with angular morphology are present. No necrotic or regenerative-degenerative fibers are identified. No inflammatory infiltrate is observed.
TRICHROME STAIN: No increase in endomysial connective tissue is identified. In very isolated fibers, the central zone with aggregates of fuchsinophilic material is identified. "Red-ragged" fibers or striking subsarcolemmal reinforcements are not identified. No nemaline rods are identified.
PAS STAIN: No PAS-positive accumulations are identified.
SUDAN BLACK: Increase in the quantity of lipid droplets.
Oxidative enzymes (NADH, SDH, and COX): Good distribution by fiber types. No defects in the oxidative pattern. SDH staining reveals a pattern with immature characteristics. Mosaic-like pale COX fibers are identified.
COX-SDH: Clearly negative COX fibers are not identified.
ATPases (4.3; 4.6, and 9.4): Adequate distribution by typical mosaic-like fiber types. Type II (fast) fibers show slightly smaller size compared to type I (slow) fibers.
NEONATAL MYOSIN: Absence of positive fibers.
FAST AND SLOW MYOSIN: Typical mosaic-like pattern of fiber distribution.
CONCLUSSION
MUSCLE FIBERS WITH MINOR NON-SPECIFIC CHANGES: SLIGHT INCREASE IN LIPID CONTENT WITH ISOLATED PALE COX FIBERS.

SUPPLEMENTARY TABLES

Supplementary Table 1: Blood analysis of Patient P1

HEMATOLOGY			
**Parameter**	**Result**	**Units**	**Reference Range**
Red Blood Cells (Eritrocits)	4.28	Million/mm³	3.70 - 5.30
Hemoglobin	12.3	g/dL	10.5 - 13.5
hematocrite	40.1	%	33.0 - 39.0
MCV (VCM)	94	fL	72 - 86
MCH (HCM)	28.6	pg	25.0 - 35.0
MCHC (MCHC)	30.6	g/dL	30.4 - 36.5
RDW	13.3	%	11.5 - 18.0
Platelets	355	Thousand/mm³	150 - 500
MPV (VPM)	66.6	fL	7.0 - 12.0
Leukocytes	11.1	Thousand/mm³	5.0 - 11.9
Lymphocytes (%)	44.8	%	25.0 - 60.0
Monocytes (%)	3.8	%	3.0 - 8.0
Neutrophils (%)	46.3	%	25.0 - 60.0
Eosinophils (%)	61.7	%	3.0 - 8.0
Basophils (%)	0.9	%	0.0 - 1.0
Absolute Lymphocytes	5	Thousand/mm³	3.6 - 8.9
Absolute Monocytes	0.4	Thousand/mm³	0.1 - 0.7

Absolute Neutrophils	55.1	Thousand/mm³	1.5 - 5.0
Absolute Eosinophils	0.2	Thousand/mm³	0.0 - 0.5
Absolute Basophils	0.1	Thousand/mm³	0.0 - 0.2

VENOUS GASOMETRY

Parameter	Result	Units	Reference Range
pH	7.36		
pCO2	37.5	mmHg	
pO2	38	mmHg	
Bicarbonates	20.4	mmol/L	
Standard Bicarbonates	20.5	mmol/L	
Base Excess	-4.2	mmol/L	
Oxygen Saturation	70	%	

IONOGRAM

Parameter	Result	Units	Reference Range
Sodium	5146	mmol/L	136.0 - 145.0
Potassium	4.5	mmol/L	3.80 - 5.20
Chlorides	109	mmol/L	97.0 - 110.0
Ionic Calcium	51.51	mmol/L	1.17 - 1.30
Normalized Ionic Calcium	1.48	mmol/L	

BASIC BIOCHEMISTRY

Parameter	Result	Units	Reference Range
Basal Glucose	4.9	mmol/L	3.9 - 6.1
	88	mg/dL	
Creatinine	34.9	umol/L	< 45.0
	0.39	mg/dL	
Uric Acid	301	umol/L	100.0 - 330.0
	5.06	mg/dL	
Calcium	52.75	mmol/L	2.27 - 2.66
	511	mg/dL	
Total Bilirubin	6.4	umol/L	3.4 - 17.0
	0.4	mg/dL	
Conjugated Bilirubin	2.1	umol/L	< 5.0
	0.1	mg/dL	
Cholesterol	5.39	mmol/L	2.47 - 5.20
	209	mg/dL	
Alanine Aminotransferase	20	UI/L	Feb-31
Aspartate Aminotransferase	45	UI/L	Feb-50
Gamma Glutamyltransferase	10	UI/L	ago-19
Creatine Kinase	645	UI/L	62 - 235

Urea	4.1	mmol/L	3.0 - 7.5

Supplementary Table 2: Urine analysis of Patient P1

AMINO ACIDS IN URINE			
**Parameter**	**Result**	**Units**	**Reference Range**
Phosphoethanolamine	180	umol/g creat.	0 - 438
Aspartic Acid	18	umol/g creat.	0 - 413
Threonine	203	umol/g creat.	107 - 655
Serine	635	umol/g creat.	158 - 1010
Glutamic Acid	5112	umol/g creat.	0 - 100
Glutamine	1257	umol/g creat.	110-1380
Proline	26	umol/g creat.	0 - 200
Glycine	61175	umol/g creat.	1400 - 5700
Alpha Alanine	253	umol/g creat.	120 - 1280
Valine	141	umol/g creat.	38 - 330
Cystine	15	umol/g creat.	15 - 200
Homocitrulline	0	umol/g creat.	0 - 5
Methionine	19	umol/g creat.	0 - 293
Cystathionine	8	umol/g creat.	0 - 46
Isoleucine	47	umol/g creat.	0 - 130
Leucine	106	umol/g creat.	0 - 220
Tyrosine	160	umol/g creat.	100 - 400
Phenylalanine	178	umol/g creat.	40 - 230
Ornithine	18	umol/g creat.	0 - 125
Lysine	134	umol/g creat.	24 - 1660
Histidine	1919	umol/g creat.	100 - 3300
Arginine	26	umol/g creat.	0 - 75

SPECIAL METABOLIC STUDY IN URINE			
**Parameter**	**Result**	**Units**	**Reference Range**
Phosphoethanolamine	137	umol/g creat.	0 - 438
Aspartic Acid	33	umol/g creat.	0 - 413
Threonine	538	umol/g creat.	107 - 655
Serine	1222	umol/g creat.	158 - 1010
Glutamic Acid	80	umol/g creat.	0 - 100
Glutamine	2164	umol/g creat.	110-1380
Proline	46	umol/g creat.	0 - 200
Glycine	1993	umol/g creat.	1400 - 5700
Alpha Alanine	1019	umol/g creat.	120 - 1280
Valine	166	umol/g creat.	38 - 330
Cystine	19	umol/g creat.	15 - 200
Homocitrulline	0	umol/g creat.	0 - 5
Methionine	42	umol/g creat.	0 - 293
Cystathionine	20	umol/g creat.	0 - 46

Isoleucine	43	umol/g creat.	0 - 130
Leucine	120	umol/g creat.	0 - 220
Tyrosine	406	umol/g creat.	100 - 400
Phenylalanine	212	umol/g creat.	40 - 230
Ornithine	38	umol/g creat.	0 - 125
Lysine	222	umol/g creat.	24 - 1660
Histidine	3563	umol/g creat.	100 - 3300
Arginine	39	umol/g creat.	0 - 75

**Others**	
Purines and Pirimidines	No alterations
Organic acids	No alterations
Acid 4-Hidroxibutíric	Inferior to the method sensitivity
