# Supplementary material for: SDS22 coordinates the assembly of holoenzymes from nascent protein phosphatase-1
Source: Nat Commun. 2024 Jun 25;15:5359. doi: 10.1038/s41467-024-49746-4 (PMC11199634; doi:10.1038/s41467-024-49746-4)
Supplement: Supplementary file 3 — Description of Additional Supplementary Files [file 41467_2024_49746_MOESM3_ESM.pdf]

<u>Description of Additional Supplementary Files</u>

Supplementary Data 1. Dataset of RNA sequencing

Supplementary Data 2. Dataset of phospho-proteomics

Supplementary Data 3. Dataset of Whole-genome sequencing of patient P1

Supplementary Data 4. The sequence of all new constructs generated for this study

Supplementary Data 5. The source of all key materials (chemicals, constructs, antibodies, siRNAs, cell lines) and adopted software used in this study
