# Supplementary material for: SDS22 coordinates the assembly of holoenzymes from nascent protein phosphatase-1
Source: Nat Commun. 2024 Jun 25;15:5359. doi: 10.1038/s41467-024-49746-4 (PMC11199634; doi:10.1038/s41467-024-49746-4)
Supplement: Supplementary file 9 — Reporting Summary [file 41467_2024_49746_MOESM9_ESM.pdf]

nature portfolio

Corresponding author(s): Mathieu Bollen

Last updated by author(s): May 15, 2024

Reporting Summary

Nature Portfolio wishes to improve the reproducibility of the work that we publish. This form provides structure for consistency and transparency in reporting. For further information on Nature Portfolio policies, see our Editorial Policies and the Editorial Policy Checklist.

Statistics

For all statistical analyses, confirm that the following items are present in the figure legend, table legend, main text, or Methods section.

n/a	Confirmed	
☐	☒	The exact sample size (*n*) for each experimental group/condition, given as a discrete number and unit of measurement
☐	☒	A statement on whether measurements were taken from distinct samples or whether the same sample was measured repeatedly
☐	☒	The statistical test(s) used AND whether they are one- or two-sided<br>*Only common tests should be described solely by name; describe more complex techniques in the Methods section.*
☒	☐	A description of all covariates tested
☐	☒	A description of any assumptions or corrections, such as tests of normality and adjustment for multiple comparisons
☐	☒	A full description of the statistical parameters including central tendency (e.g. means) or other basic estimates (e.g. regression coefficient) AND variation (e.g. standard deviation) or associated estimates of uncertainty (e.g. confidence intervals)
☐	☒	For null hypothesis testing, the test statistic (e.g. *F*, *t*, *r*) with confidence intervals, effect sizes, degrees of freedom and *P* value noted<br>*Give P values as exact values whenever suitable.*
☒	☐	For Bayesian analysis, information on the choice of priors and Markov chain Monte Carlo settings
☒	☐	For hierarchical and complex designs, identification of the appropriate level for tests and full reporting of outcomes
☒	☐	Estimates of effect sizes (e.g. Cohen's *d*, Pearson's *r*), indicating how they were calculated

Our web collection on statistics for biologists contains articles on many of the points above.

Software and code

Policy information about availability of computer code

Data collection	- LuminoSkan Ascent Software (Thermo Fisher Scientific) was used for measurement of luciferase activity.<br>- PyMOL (Schrödinger) and UCSF ChimeraX (National Institutes of Health) were used to show the protein structural model.<br>- ImageJ (National Institutes of Health) and LAS AF Lite (Leica) were used for collection of Immunofluorescence images and live cell imaging.<br>- ImageQuant LAS 4000 Control Software (GE Healthcare) was used to measure horseradish peroxidase (HRP) luminescence following<br>- Immunoblotting with HRP-tagged secondary antibodies.<br>- BD Canto II flow cytometer was used for the collection of Flow Cytometry data.
Data analysis	- Microsoft Excel (Microsoft) was used for data processing, including normalization.<br>- GraphPad Prism 9.4.0 was used for statistical analysis (GraphPad Software).<br>- FlowJo software was used for the analysis of Flow Cytometry data<br>- MicroCal iTC200 for the analysis of Isothermal titration calorimetry data

For manuscripts utilizing custom algorithms or software that are central to the research but not yet described in published literature, software must be made available to editors and reviewers. We strongly encourage code deposition in a community repository (e.g. GitHub). See the Nature Portfolio guidelines for submitting code & software for further information.

Data

Policy information about availability of data

All manuscripts must include a data availability statement. This statement should provide the following information, where applicable:

- Accession codes, unique identifiers, or web links for publicly available datasets
- A description of any restrictions on data availability
- For clinical datasets or third party data, please ensure that the statement adheres to our policy

The primary datasets of all illustrations were deposited at Figshare (Figshare DOI https://doi.org/10.6084/m9.figshare.25533646). The AlphaFold models of SDS22:PP1:I3 are available in ModelArchive with the access code: W6u2MgaTPM [https://www.modelarchive.org/doi/10.5452/ma-tmp13]. The RNA sequencing data generated in this study were deposited in the GEO database with the access code: glixmqswlhevlcd [https://www.ncbi.nlm.nih.gov/geo/query/acc.cgi?acc=GSE249519]. Raw MS data for this study are available at ProteomeXchange with the access code: p211 [https://massive.ucsd.edu/ProteoSAFe/dataset.jsp?task=7fa2a943422148c19d91e017a0caf990]. All remaining data can be found in the Article, Supplementary, or figshare files.

Research involving human participants, their data, or biological material

Policy information about studies with human participants or human data. See also policy information about sex, gender (identity/presentation), and sexual orientation and race, ethnicity and racism.

Reporting on sex and gender	We have obtained clinical and genetic data from 1 female patient of 10 years.
Reporting on race, ethnicity, or other socially relevant groupings	caucasian
Population characteristics	n/a
Recruitment	Patient P1 was enrolled in the URDCat (Undiagnosed Rare Diseases in Catalonia) project. Her parents provided written informed consent for genetic testing and release of clinical data.
Ethics oversight	The UZ hospital ethical committee gave consent to use the patient dataset for publication purposes.

Note that full information on the approval of the study protocol must also be provided in the manuscript.

Field-specific reporting

Please select the one below that is the best fit for your research. If you are not sure, read the appropriate sections before making your selection.

☒ Life sciences ☐ Behavioural & social sciences ☐ Ecological, evolutionary & environmental sciences

For a reference copy of the document with all sections, see nature.com/documents/nr-reporting-summary-flat.pdf

Life sciences study design

All studies must disclose on these points even when the disclosure is negative.

Sample size	No statistical methods were used to predetermine sample size. Rather, the selection of this sample size was informed by preliminary experiments that suggested its adequacy for detecting significant differences in the mean.
Data exclusions	No data were excluded.
Replication	Each assay was replicated multiple times, using a combination of technical replicates (using the same set of lysates for split-luciferase assays and same cells seeding at 3 different wells for IncuCyte live-cell assays) and biological replicates. The number of technical replicates and biological replicates (independent experiments) are indicated in the figure legends.
Randomization	The experiments were not randomized
Blinding	The Investigators were not blinded to allocation during experiments and outcome assessment

Reporting for specific materials, systems and methods

We require information from authors about some types of materials, experimental systems and methods used in many studies. Here, indicate whether each material, system or method listed is relevant to your study. If you are not sure if a list item applies to your research, read the appropriate section before selecting a response.

Materials & experimental systems

n/a	Involved in the study	
☐	☒	Antibodies
☐	☒	Eukaryotic cell lines
☒	☐	Palaeontology and archaeology
☒	☐	Animals and other organisms
☒	☐	Clinical data
☒	☐	Dual use research of concern
☒	☐	Plants

Methods

n/a	Involved in the study	
☒	☐	ChIP-seq
☐	☒	Flow cytometry
☒	☐	MRI-based neuroimaging

Antibodies

Antibodies used

The source of the used antibodies was as follows:
SDS22 (Santa Cruz, 612548), GAPDH (Cell Signaling Technology, 2118),
phospho-Ezrin(T567)/Radixin(T564)/Moesin(T558) (Cell Signaling Technology, 3141),
alpha-tubulin (Sigma-Aldrich, T6074),
beta-actin (Santa Cruz, sc-69879),
histone H3 (Sigma-Aldrich, h0614), GFP (Santa Cruz, sc-9996),
Inhibitor 3 (Eurogentec, SY0193),
phosphoserine/threonine (BD Transduction Laboratories, 612548),
MYPT1 (Santa Cruz, sc-514261),
CDCA2(Repoman) (Sigma-Aldrich, HPA030049),
MAD2 (Bethyl laboratories Inc, A300-301A),
BCLAF1 (Bethyl laboratories Inc, A300-610A),
PP1 gamma (Santa Cruz, sc-515943),
Flag (Sigma-Aldrich, F1804),
The monoclonal anti-PP1 antibody was purified on protein-A Sepharose CL-4B (GE Healthcare).

Antibodies for immunofluorescence:
Donkey anti-Rabbit: Alexa Fluor 555 (Invitrogen - ThermoFisherScientific, 2088692)
Goat anti-Mouse: Alexa Fluor 633 (Invitrogen - ThermoFisherScientific, A21052)

Validation

Antibodies were validated by the manufacturer and/or were used extensively in previously published studies, including studies from our group.

Eukaryotic cell lines

Policy information about cell lines and Sex and Gender in Research

Cell line source(s)

HCT116 Tet-OsTIR1 was Obtained from Riken Cell Bank, Japan,
HEK293T was obtained from ATCC (CRL-11268 293T/17),
HeLa Flp-In T-REx Host cell line was recieved from Prof.D.Taylor, Manchester Uni, UKHeLa,
Flp-In T-REx (Stephen Taylor) - Clover-PP1g was from previous work in host laboratory,
HeLa Flp-In T-REx (Stephen Taylor) - Clover-PP1g (K147A/K150A) was from previous work in host laboratory,
HeLa Kyoto - H2B mRFP/mEGFP α tubulin was recieved from Prof.D.Gerlich, Vienna Biocenter,
HeLa FLpIn T-REx PP1gamma-2x Strep was recieved from Prof.H. Meyer, Duisburg-Essen Uni, Germany

Authentication

For HEK293T cells no authentication was performed since its purchase from supplier. Other cell lines were authenticated in previous studies.

Mycoplasma contamination

Mycoplasma negative

Commonly misidentified lines
(See ICLAC register)

No commonly misidentified cell lines were used in this study.

Plants

Seed stocks: n/a

Novel plant genotypes: n/a

Authentication: n/a

Flow Cytometry

Plots

Confirm that:

- [x] The axis labels state the marker and fluorochrome used (e.g. CD4-FITC).
- [x] The axis scales are clearly visible. Include numbers along axes only for bottom left plot of group (a 'group' is an analysis of identical markers).
- [x] All plots are contour plots with outliers or pseudocolor plots.
- [x] A numerical value for number of cells or percentage (with statistics) is provided.

Methodology

Sample preparation: For cell-cycle analysis with flow cytometry, cells were harvested by trypsinization, washed with PBS, and fixed overnight with 70 % ethanol at -20°C. Subsequently, the cells were washed twice with PBS, resuspended in PBS containing 0.05 % Triton and 0.1 mg/ml RNAse, and incubated for 30 minutes at 37°C. Propidium iodide was added at a final concentration of 70 µg/ml.

Instrument: Canto II AIG

Software: BD Canto II flow cytometer, FlowJo software

Cell population abundance: 50.000-100.000 cells/assay

Gating strategy: For cell cycle graphs needed:
FSC-A vs SSC-A
PerCP-Cy5-5-A histogram

- [ ] Tick this box to confirm that a figure exemplifying the gating strategy is provided in the Supplementary Information.
